# The feasibility and efficacy of secondary neck dissections in thyroid cancer metastases

**DOI:** 10.1007/s00405-013-2588-8

**Published:** 2013-06-16

**Authors:** Malgorzata Wierzbicka, Edyta Gurgul, Elżbieta Wasniewska-Okupniak, Maria Gryczynska, Tomasz Piorunek, Marek Ruchala

**Affiliations:** 1 Department of Otorhinolaryngology, Head and Neck Surgery, Poznan University of Medical Sciences, Przybyszewskiego Street 49, 60-355 Poznan, Poland; 2 Department of Endocrinology, Metabolism and Internal Diseases, Poznan University of Medical Sciences, Przybyszewskiego Street 49, 60-355 Poznan, Poland; 3 Department of Pulmonology, Allergology and Respiratory Oncology, Poznan University of Medical Sciences, Szamarzewskiego Street 84, 60-569 Poznan, Poland

**Keywords:** Thyroid cancer, Nodal metastases, Neck dissection

## Abstract

The purpose of the study was to assess the feasibility of secondary neck dissections (ND) in different types of thyroid cancer (TC), to evaluate the influence of ND extent on morbidity and to describe biochemical and clinical outcomes. 51 patients previously operated for TC (33-well differentiated TC-WDTC, 15 medullary TC-MTC, 3 poorly differentiated TC-PDTC) presenting detectable nodal disease. Reoperations covered I–VII neck levels. Radical neck dissection was performed in 22 patients, selective neck dissection in 29 patients. 14 central compartment (CC), 10 mediastinal and 41 level IV excisions were performed. Postoperative complications occurred in 13 patients: 4 chyle leaks, 3 massive bleedings, 8 permanent vocal cord pareses, hypoparathyroidism in 22 patients (43.1 %), 2 patients expired in perioperative period. In WDTC: in seven patients thyroglobulin level normalized directly after ND, in ten patients in the follow-up; six patients developed distant metastases. None of the patients with MTC achieved calcitonin level <10 pg/ml; nine patients developed distant metastases. None of the patients with PDTC achieved Tg <2 mg/ml; two patients died, the third developed distant metastases. Secondary ND in TC present a challenge by means of surgical approach and possibility of complications. In MTC and PDTC the long-term results were unsatisfactory. In WDTC, the secondary ND should be performed due to strong indications. Metastases localization in levels IV, VI, VII were connected with high complication rate, but these surgeries were crucial for satisfactory oncological outcomes.

## Introduction

Secondary surgery of the neck for persistent or recurrent thyroid cancer (TC) is still the subject of debate [1]. The advocated extent of the salvage neck dissection (ND) has not been established yet. Subsequent procedure for the central compartment (CC) carries the risk related directly to the anatomy of this region. Upper mediastinum (level VII) and supraclavicular fossa (level IV) remains challenging even in the primary approach. In patients with TC, the balance between the risk of morbidity from secondary ND and the risk of untreated cancer disease still remains an unresolved issue [2]. Previous articles have evaluated the feasibility and efficacy of reoperations in thyroid cancer (in persistent or recurrent disease), but authors had focused on well differentiated thyroid cancer (WDTC) and surgeries limited to the central compartment [3–5]. In most cases, thyroid cancer recurrences in cervical lymph nodes can be visualized with ultrasound examination, what helps to arrange further therapeutic procedures [6].

The aim of the study was (1) to assess the feasibility of secondary neck dissections (ND) in well differentiated thyroid cancer, medullary cancer (MTC) and poorly differentiated thyroid cancer (PDTC), (2) to evaluate the influence of ND extent on morbidity and (3) to describe the biochemical and clinical outcomes with special regard to lower neck levels (IV, VI, VII).

## Materials and methods

The prospective study was conducted in a tertiary university centre in 2003–2011. The study group consisted of 51 consecutive patients with persistent/recurrent thyroid cancer (32 females and 19 males). All patients enrolled to the study had been previously operated for TC in 11 different general surgery units and then followed-up in the Department of Endocrinology, Metabolism and Internal Medicine of Poznan University of Medical Sciences. Treatment failures had been subsequently referred to the Department of Otorhinolaryngology, Head and Neck Surgery, Poznan Medical University. All patients had clinically detectable nodal disease. The recurrent or persistent thyroid cancer neck disease was defined as: positive imaging on ultrasound, computed tomography or radioiodine whole body scan, increased thyroglobulin (Tg) or calcitonin levels.

The age of the patients at the onset of disease ranged from 16 to 76 years (mean 48 years). 46 patients had previously undergone one surgical procedure for TC and 5 patients two or more. Histological appearance of the primary lesion was determined according to the World Health Organization histological classification and was as follows: 33 cases of WDTC, 15 cases of MTC, 3 cases of PDTC. Staging was performed according to the American Joint Committee on Cancer Classification (AJCC) Cancer Staging Manual revised in 2009 [7]. The advancement of the primary tumor was as follows: T1 in 6 cases, T2 in 16 cases, T3 in 18 cases and T4 in 11 cases. Nodal disease at the first presentation was N0 in 36 (60.8 %), and N+ in 15 (39.2 %) patients respectively. The time between the primary surgery and reoperation ranged from 7 months to 16 years (median 2 years).

Thyrotropin (TSH) stimulation had shown increased Tg level (>2 ng/ml) in 31/33 patients with WDTC. The values ranged from 2.32 to >900 ng/ml. The supraphysiological thyroid hormone replacement was introduced in this group to achieve TSH suppression (<0.1 U/ml). In all patients with medullary cancer calcitonin level was increased (>10 pg/ml) and ranged from 13 pg/ml to over scale measurements >480 pg/ml.

The indirect laryngoscopy and stroboscopy were performed before and after the reoperation (on the 1st, 7th day and 6 months later if needed). Seven patients had had unilateral vocal cord paralysis prior to secondary ND.

The mean follow-up time after the neck reoperation was 3.4 ± 2.3 years.

All reoperations were carried out with therapeutic intent. The reoperative surgery covered I–VII neck levels. The choice of the procedure depended on rN advancement, macrometastases location and tumor histology.

The Radical Neck Dissection (RND) included the lateral node compartment: jugular chain nodes (levels I, II, III, IV), posterior neck (level V), from the skull base to the subclavian vein and laterally to the trapezius muscle. In all cases the accessory nerve was intentionally preserved. The sternocleidomastoid muscle and jugular vein were dissected in strictly chosen cases (in patients with lymph nodes metastases >3 cm adjacent to the vessel wall). Selective Neck Dissection (SND) most often cleared levels IIa, III and IV. Dissection of the central compartment (CC) included level VI nodes extended vertically from the hyoid bone to the thoracic inlet and horizontally between the carotid sheaths (prelaryngeal, pretracheal and paratracheal nodes). Level VII included upper mediastinal nodes––from the sternum to the brachio-cephalic vein.

Student’s *t* test was used for statistical analysis of continuous variables. Categorical variables were analyzed with Fisher’s exact test and the χ^2^ test. *P* < 0.05 was used to define statistical significance. The project was carried out with the approval of the Local Ethics Committee.

## Results

The extension of the secondary ND and patients data concerning neck relapse are presented in Table 1.

**Table 1:** The types of thyroid cancer, localization of the excised nodes and the complications of secondary neck dissection

	No.	Follow up time (years)	No. of complications		
			RLN	HP	Other
WDTC	33	2.6	8	12	6
Medullary cancer	15	1.3	3	8	3
Poorly differentiated thyroid cancer	3	1.3	2	2	2
Neck levels I–III only	6	2.7	2	2	0
Neck level VI, VII only	2	1	1	1	1
Neck level IV only	1	5	0	0	0
Neck level IV, VI, VII+ other	14	1.1	6	8	5
**Total**	**51**	**1.8**	**13**	**22**	**11**

*RLN* recurrent laryngeal nerve injury (both persistent and transient), *HP* persistent hypoparathyroidism

RND was performed in 22 patients (43.1 %; 15-WDTC, 6-MTC, 1-PDTC) and SND in 29 patients (56.9 %; 18-WDTC, 9-MTC, 2-PDTC). CC (level VI) was cleaned in 14 patients (27.5 %; 9-WDTC, 4-MTC, 1-PDTC)––in two as the main procedure and in 12 as complementary to lateral node compartment dissection. Level VII (upper mediastinal nodes) were dissected in 10 patients (19.6 %; 7-WDTC, 1-MTC, 2-PDTC), levels I, II, III, IV and V in 3, 36, 40, 41 and 21 patients respectively. All together lower neck reoperation (IV, VI, VII) was performed in 46 cases (90.2 %). 11 patients were operated more than once (4 patients with WDTC, 6-MTC, 1-PDTC). Total amount of neck dissections was 66.

In our study group the localization of recurrence (level I–III, IV, V, VI, VII) and the time between the primary surgery and the recurrence did not depend on patients gender, age or the type of thyroid cancer. In three patients very aggressive course of the recurrent disease was observed: in two cases gross infiltration of the larynx and esophagus requiring total laryngectomy with partial esophagectomy. Third patient required tracheal resection.

### Histopathological findings

The number of removed lymph nodes ranged from 1 to 42. In the majority of patients metastatic TC was present in multiple nodes. Data of the neck dissection specimens including number of metastatic nodes end extracapsular spread (ECS) was presented in Table 2.

**Table 2:** Histopathological results of the neck dissection specimen

Neck level	No. of nodes removed	No. of patients	pN+	ECS
I/II/III only	34	6	8	0
IV only	1	1	1	1
IV, VI, VII + other	167	14	94	6
VI, VII only	11	2	10	1

*ECS* extra capsular spread

The number of metastatic nodes ranged from 1 to 28. Single metastatic lymph nodes were found in 11 patients (21.6 %), 2 metastatic lymph nodes in 10 patients (19.6 %), three in 1 patient (2 %), 4 in 3 patients (5.9 %) and 5 or more in 25 patients (47.1 %). The metastatic spread was predominantly located in the lower neck. Metastases were confirmed in all 14 CC and all 10 mediastinal specimens; thus in levels VI and VII there were no false negative results. Out of 59 lateral neck specimens metastases were found in the levels II and III in 37 cases (72 %) and in the level IV in 35 cases (68 %). Bilateral neck metastases were found in 17 patients (33.3 %). The occurrence of bilateral metastases did not depend on the type of thyroid cancer, but was often observed in younger patients (*p* = 0.07) and in patients with primary surgery performed at younger age (*p* = 0.049). Furthermore, these two groups more frequently required radical neck dissection (*p* < 0.01 in both groups). In six specimens from the VIIth level the thymus tissue was found. In 43 procedures (84 %) free surgical margins were obtained (27-WDTC, 15-MTC, 1-PDTC).

### Complications and morbidity

The technical difficulties included disturbed anatomy, scars, infiltration of subclavicular vessels or of the junction between subclavian and internal jugular vein (5 patients), infiltration of the larynx or esophagus (2 patients), infiltration of trachea (1 patient).

Postoperative vocal cord paresis was observed in 13 (25.5 %), transient in 5 (9.8 %), permanent in 8 (15.7 %) patients. In seven patients recurrent laryngeal nerve (RLN) was injured due to direct tumor invasion––in three (MTC) nerve was intentionally sectioned and in four (WDTC) nerve was detached from the tumor. In six patients RLN visualization was impossible due to rigidity of the scar tissue; paresis occurred postoperatively. Six patients suffered from severe dyspnoea: in three laterofixation and in three emergency tracheotomy was indispensable. Permanent tracheostomy was maintained in five (9.8 %) patients: in three with vocal cord paresis and in two after laryngectomy.

Other complications were observed in seven patients (13.7 %). Four patients had postoperative chyle leakage: in two cases low output, stopped after conservative treatment after 6–8 days, in one patient high-output leak was controlled on the 5th day by direct ligation. In the 4th case the patient suffered from chylothorax, required two reoperations, that occurred unsuccessful and the patient expired on the 36th day. Massive bleeding occurred intraoperatively in three patients (in one from common carotid artery, in two from the stump of internal jugular vein). One patient died due to massive bleeding from common carotid artery on the 2nd day after surgery.

The postoperative PTH serum level was within the normal range (15–65 pg/ml) in 30 (58.8 %) patients. However, hypocalcaemia requiring vitamin D and/or calcium supplementation appeared in 22 patients (43.1 %). It is important to note, that 8 out of 14 patients operated on IV, VI, VII levels developed hypoparathyroidism (57 %).

Horner syndrome was not observed in the study group.

There was no correlation between the rate of complications and patients age, gender, time from primary surgery or cancer histology. Furthermore, the number of dissected lymph nodes and the presence of bilateral metastatic changes also did not influence significantly the frequency of complications. Localization was a crucial morbidity factor. Secondary ND performed in the left subclavian or in the upper mediastinal area were connected with gross complications: 5/8 cases of RLN paresis, 2 chyle leakage and 1 chylothorax and 2 massive bleedings.

### Outcomes of secondary nodal surgery

Two of the patients had perioperative complications and expired soon after the procedure (2nd and 36th day)––both with WDTC.

Out of the other patients with WDTC: one patient died of disseminated TC 2 years after neck dissection, at age of 74 years; two patients (6 %) developed new recurrence in the upper mediastinum, not qualifying for the additional ND, 5 patients (15.2 %) developed lung metastases and one bone metastases (3 %).

Seven patients (21.2 %) indicated biochemical cure at the first control examination after the reoperative surgery (Tg level <2 ng/ml evaluated on thyrotropin stimulation), in ten patients Tg was <2 ng/ml in the follow-up (30.3 %), in six patients (18.2 %) Tg level decreased after operation. Seven patients (21 %) still presented with notably elevated Tg levels >30 ng/ml.

None of the patients with MTC achieved calcitonin level lower than 10 pg/ml. Nine patients developed distant metastases in 10–16 months time (lung metastases in 7 patients, bone metastases in 3 patients). Four patients expired in the follow-up.

None of the patients with poorly differentiated thyroid cancer achieved Tg level <2 ng/ml). One patient developed disseminated lung and bone metastases and is still under control of our clinic (4 years after neck dissection). Two other patients died.

Distant metastases were observed more frequently in patients with MTC and PDTC (*p* = 0.007).

## Discussion

The American Thyroid Association (ATA) and National Comprehensive Cancer Network (NCCN) recommends treatment options for recurrent or persistent WDTC: radioiodine therapy for radioiodine sensitive disease and surgery for clinically significant disease. The surgery is recommended for curative intent and to prevention of locoregional complications [8]. Secondary surgery in CC has been approved in the literature [4, 9, 10], although is associated with high risk of complications and moderate possibilities of tumor eradication. Therefore, the question is, how to obtain the balance between the cure and complication rates and whether the patient will benefit from the surgery. In patients with medullary cancer the salvage surgery is still the treatment of choice.

The sites frequently involved in recurrent or persistent disease include retrocarotid nodes, nodes deep to laryngeal recurrent nerve, retroclavicular nodes, retrosternal nodes and nodes running along the superior thyroid vascular pedicle medial to the carotid bifurcation [11]. Leaving positive nodes in tracheoesophageal groove may expose the patient to risk of tracheal, esophageal or even laryngeal invasion [12]. Laryngeal and esophageal invasion was observed by Clayman et al. [4] in 3/63 patients; in our group 2/51 patients definitely needed total laryngectomy. Roh et al. [13] found nodal metastases in central and bilateral central compartment in 86.7 and 42.2 % cases respectively but little attention was devoted to the neighboring IV level. Metastasizing pattern in our group revealed pN+ in level IV, VI and VII in 41, 16 and 10 patients respectively; in the IVth level over 70 %, in CC 100 % of taken nodes were pN+; 17 % of patients had metastases bilaterally. Ahmadi et al. [3] comparing primary and recurrent disease distribution stated, that there was no difference in frequency in levels I, II, III and V but level IV was more common in the recurrent cases. Salvage neck surgery in our group confirmed the necessity of clearing all lower neck basins. In our group younger patients more frequently required RND (*p* < 0.01).

It is important to confront the surgical feasibility and morbidity in patients with recurrences in the lower neck anatomical region. The previous studies demonstrated that there is no additional morbidity in secondary procedures and complication rate is similar to initial surgical procedure, if performed by experienced surgeon [2, 14]. Numerous authors described the CC salvage surgery as safe and efficient oncological procedure exhibiting minimal morbidity [1, 4, 5, 10, 15]. On the other hand, Hartl and Travagli [11] underlined additional morbidity when central ND is performed as secondary procedure. Although the boundaries and the anatomical structures that require dissection are the same, the scar tissue and neovascularization complicates the access to this region; the sternohyoid and sternothyroid muscles are adherent to the trachea in dense scar tissue. Localization of RLN nerve may be particularly difficult in the widespread fibrosis [10]. Thus, reoperative surgery of the CC is associated with higher risk of vocal folds paresis. According to Mirghani et al. [16] CC ND can be performed with minimal morbidity, when recurrent laryngeal nerve is not invaded. In our group in 3/15 medullary cancers the nerve was intentionally sectioned, in 4/33 WDTC detached from the tumor. In eight cases (15.7 %) permanent vocal cord paresis was observed; in three (5.9 %) tracheotomy had to be maintained.

It has been stated, that parathyroid glands are at increased risk for injury and permanent hypoparathyroidism is the most common adverse event in CC secondary surgery [2]. Permanent hypoparathyroidism has been reported to range between 2.7 [5] and 9 % [16]. In our study long-term follow-up revealed permanent hypoparathyroidism in 22 patients (43.1 %).

The outcomes of reoperative surgery for WDTC can be categorized as the absence of biochemical disease or clinically detectable disease. In our center the required Tg level assessed on TSH stimulation was determined to be lower than 2 ng/ml. According to Hughes et al. [1] biochemical complete response obtained in 26 % of patients may be concentrated on locoregional control of disease to prevent complications due to local invasion rather than elimination of biochemically active cancer. In our study stimulated Tg <2 mg/ml was observed in seven patients with WDTC directly after reoperative procedure (21.2 %) and in ten patients in the follow-up (30.3 %). Six patients with WDTC developed distant metastases.

In MTC or PDTC the final results of secondary surgery were poor. None of the patients with MTC achieved calcitonin level lower than 10 pg/ml and none of the patients with PDTC achieved biochemical remission. It should be therefore concluded, that MTC and PDTC need more rigorous preoperative work up to ensure that distant metastases are excluded, otherwise the prognosis will be unfavorable despite successful neck dissection.

Our study reports the large cohort of patients undergoing secondary ND due to thyroid cancer. Surgical approach and possibility of complications in this group present a challenge. In MTC and PDTC the long-term results were unsatisfactory (calcitonin/Tg still elevated, frequent distant metastases). In WDTC the secondary neck dissection should be performed in cases in which there are strong indications for surgical procedure. Metastases localization in levels VI, VII, IV was connected with high complication rate. On the other hand total clearance of these basins proved to be crucial for the satisfactory oncological outcomes.
